# Safety Profile of Zavegepant in the Treatment of Acute Migraine: Insights from the FDA Adverse Event Monitoring System Database

**DOI:** 10.3390/ph19060943

**Published:** 2026-06-15

**Authors:** Giuseppe Cicala, Maria Antonietta Barbieri, Giulia Russo, Rosella Ciurleo, Rosario Grugno, Angelo Quartarone, Edoardo Spina

**Affiliations:** 1 Department of Clinical and Experimental Medicine, University of Messina, 98122 Messina, Italy; gcicala@unime.it (G.C.); giuliarusso.ab@gmail.com (G.R.); espina@unime.it (E.S.); 2 IRCCS Centro Neurolesi “Bonino-Pulejo”, Via Palermo S.S. 113, Contrada Casazza, 98124 Messina, Italy; rossella.ciurleo@irccsme.it (R.C.); rosario.grugno@irccsme.it (R.G.); angelo.quartarone@irccsme.it (A.Q.)

**Keywords:** adverse events, calcitonin gene-related peptide receptor antagonists, migraine, pharmacovigilance, zavegepant

## Abstract

**Background/Objectives**: The recent approval of the first intranasal calcitonin gene-related peptide receptor antagonist (CGRP-RA), zavegepant, has increased the relevance of this drug class in treating acute migraine. However, introducing an alternative delivery method may result in a different real-world safety profile. Thus, the aim of this study was to assess adverse events (AEs) related to zavegepant through a retrospective pharmacovigilance disproportionality analysis. **Methods**: We analyzed Individual Case Safety Reports (ICSRs) presenting zavegepant as the suspected drug, submitted to the Food and Drug Administration (FDA) Adverse Event Monitoring System (AEMS) database between 1 January 2023 and 31 December 2025. ICSRs were assessed by using descriptive and disproportionality analyses. Reporting odds ratios (RORs) with 95% confidence intervals (CIs) were used as disproportionality measures. Results were deemed significant if the ROR 95% CI lower bound was >1 and ≥3 ICSRs were available for each drug–event pair. **Results**: A total of 509 zavegepant-related ICSRs were identified. Most ICSRs involved female patients (*n* = 353; 69.4%), with a median (quartile 1, Q1–quartile 3, Q3) age of 45 (34–56) years. The Medical Dictionary for Regulatory Activities (MedDRA^®^) Preferred Terms with the highest RORs were nasal discomfort (*n* = 62; ROR = 298.85; 95%CI [228.91, 390.17]), rhinalgia (10; 126.09; [67.34, 236.09]), dysgeusia (147; 94.72; [78.19, 114.75]), pharyngeal ulceration (3; 79.20; [25.42, 246.75]), and upper-airway cough syndrome (16; 62.87; [38.19, 103.49]). **Conclusions**: These results suggest a safety profile for zavegepant consistent with previous knowledge regarding CGRP-RAs. However, nasal and/or oropharyngeal AEs, plausibly related to intranasal exposure, may affect perceived tolerability and timely use, warranting further investigation.

## 1. Introduction

Migraine is a complex neurological disorder with a polygenic and dysautonomic background [1]. Its estimated global prevalence of approximately 14% [2], corresponding to more than one billion affected individuals, places it among the most common disabling neurological conditions. Furthermore, owing to its substantial impact on quality of life, migraine is ranked as the sixth leading cause of years lived with disability worldwide [3], remarking its status as a persisting public health challenge [4]. Given its complex background, over the last 50 years, several theories have been developed to explain the mechanisms underlying migraine. Although a complete understanding of the causes underlying this condition has yet to be achieved, the activation of the trigeminovascular system is widely accepted as a key component of the pathophysiological mechanism [5,6].

The trigeminovascular system is an integrated neurovascular network composed of the trigeminal sensory neurons and their projections to cranial vessels, including meningeal and cerebral vasculature [5,7,8]. Functionally, this system mediates cranial nociception and neurovascular regulation, serving as the main pathway for migraine pain transmission [9,10,11]. However, migraine can lead to its pathological activation. Indeed, this condition has been associated with vasodilation, plasma extravasation, and meningeal inflammation [12,13]. In particular, increased levels of vasoactive neuropeptides, including calcitonin gene-related peptide (CGRP), appear to play a key role in this phenomenon and consequently in the genesis of migraine attacks when released in the meninges, triggering sterile inflammatory processes [14,15,16,17,18]. Given the importance of its action, inhibition of CGRP has gradually become a prime pharmacological target for treating migraine [19].

Today, two drug classes that are based on the blockage of the CGRP signalling pathway are available on the market. One is represented by monoclonal antibodies targeting either CGRP directly or its receptor. Thanks to their relatively high half-life, anti-CGRP monoclonal antibodies have become a relevant option for prophylactic treatment of migraine [20]. Among those, galcanezumab has also obtained FDA approval for the acute treatment of cluster headache, but not for acute migraine [21].

The other class comprises small-molecule CGRP receptor antagonists (CGRP-RAs), commonly known as gepants. CGRP RAs were the first drugs to be developed with the goal of using CGRP inhibition to treat migraine. However, during the development of the first-generation CGRP-RAs (i.e., telcagepant), major hepatotoxicity concerns emerged [22]. These issues were addressed with the introduction of the second-generation CGRP-RAs (i.e., ubrogepant, rimegepant, and atogepant). Second-generation CGRP-RAs have demonstrated their efficacy in the acute treatment of migraine, in the case of ubrogepant, in its long-term prevention in the case of atogepant, and for both indications in the case of rimegepant [23]. However, until recently, these drugs could only be administered orally. To overcome the limitations of this administration route, the third-generation CGRP-RAs have been developed. In particular, zavegepant, approved by the United States Food and Drug Administration (FDA) on 9 March 2023, as a nasal spray for the acute treatment of migraine with or without aura in adults, is the first of these novel drugs that can be administered using the intranasal route [24].

Given the fast-acting nature of the intranasal formulation, this drug could be an important tool for the acute treatment of migraine. However, this route of administration may influence the safety profile of CGRP-RAs in ways that are not yet fully understood, particularly with respect to local tolerability. Although clinical trials have established the efficacy and safety of zavegepant [25,26], they may not fully capture rare or route-specific adverse effects. Moreover, real-world safety data on zavegepant remain limited. In this context, pharmacovigilance investigations constitute a well-established and valuable approach to more comprehensively characterize the safety profile of newly marketed medications.

Thus, the aim of this study was to assess the currently available real-world adverse event (AE) data for zavegepant and to eventually identify previously unknown signals of disproportionate reporting (SDRs).

## 2. Results

### 2.1. Descriptive Analysis

**Figure 1:**
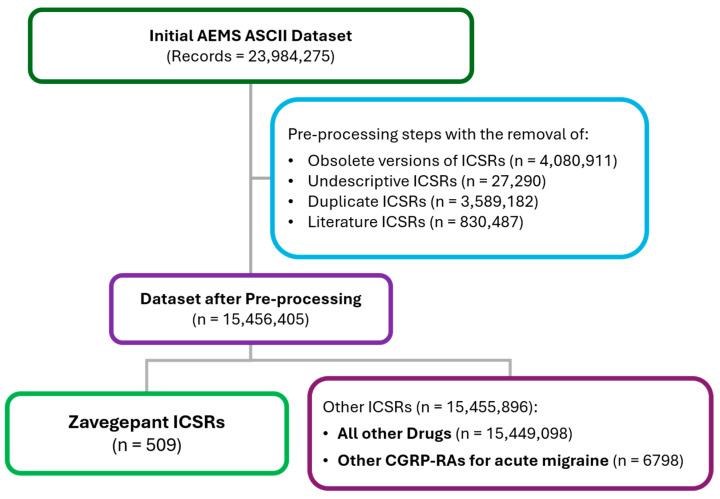
Database pre-processing and data extraction flowchart. ICSRs: Individual Case Safety Reports; AEMS: (FDA) Adverse Event Monitoring System; CGRP-RAs: calcitonin gene-related peptide receptor antagonists.

The initial dataset comprised 23,984,275 records. After dataset pre-processing (details available in Section 4) and the removal of Individual Case Safety Reports (ICSRs) identified as duplicates, 15,456,405 records remained. From these, a total of 8841 ICSRs in which a single CGRP-RA was reported as the suspected drug were extracted. After that, ICSRs reporting migraine prevention as the use indication for the CGRP-RA, as well as migraine-related Preferred Terms (PTs) as AEs, were removed. This brought the total of examined ICSRs to 7307. Of those, 509 concerned zavegepant, whereas 6798 concerned other CGRP-RAs approved for the treatment of acute migraine (ubrogepant and rimegepant) (see Figure 1 for further details).

The demographic characteristics of zavegepant-related ICSRs were broadly consistent with those of Reference Group 2 (RG2; ubrogepant and rimegepant) in terms of sex distribution. Female patients accounted for most reports in both groups (zavegepant: *n* = 353, 69.4%; RG2: *n* = 4574, 67.3%). In terms of age distribution, the majority of zavegepant-related ICSRs reported missing data (*n* = 328, 64.4%). Among reports with available age information, the median (quartile 1, Q1–quartile 3, Q3) age observed in zavegepant-related ICSRs was lower than that observed in RG2-related ICSRs (45 years, Q1–Q3 [34–56] vs. 50 years, Q1–Q3 [37–61]). The 35–64-year age group was the most represented among ICSRs with available age information in both groups (zavegepant: *n* = 117, 23.0%; RG2: *n* = 2312, 61.1%). In terms of reporter type, patients/consumers represented the reporter category with the highest frequency of observation in both ICSR groups (zavegepant: *n* = 208, 40.9%; RG2: *n* = 5254, 34.0%). It should be noted that almost all the observed ICSRs were issued to the Food and Drug Administration (FDA) Adverse Event Monitoring System (AEMS) platform via pharmaceutical companies (*n* = 503; 98.8%), whereas those uploaded directly by the FDA represented only a small minority (*n* = 6; 1.2%). The absolute majority of ICSRs related to both zavegepant (*n* = 507; 99.6%) and RG2 (*n* = 6609; 97.2%) were issued from the USA territory, see Supplementary Table S1 for further details. Considering zavegepant-related ICSRs in terms of AEs seriousness, only 30 reports (5.9%) included at least one AE classifiable as serious. Among these, most were classified as other serious important medical events (*n* = 24; 4.7%) (see Table 1 for further details).

As far as time to onset (TTO) data were concerned, those were reported in 62 (12.2%) of zavegepant ICSRs and described an onset of the observed AEs in less than 24 h in all cases. Dechallenge data were reported in 54 ICSRs (10.6%), with a positive dechallenge in 51 (10.0%) and a negative one in three (0.6%); a positive rechallenge was documented only in one case. ICSRs describing serious AEs, TTO data were available for eight cases (26.7%) and indicated an AE onset within less than 24 h in all of them. Dechallenge data were available in six serious ICSRs, describing a positive dechallenge in all of them. No rechallenge data for serious AE cases were available.

**Figure 2:**
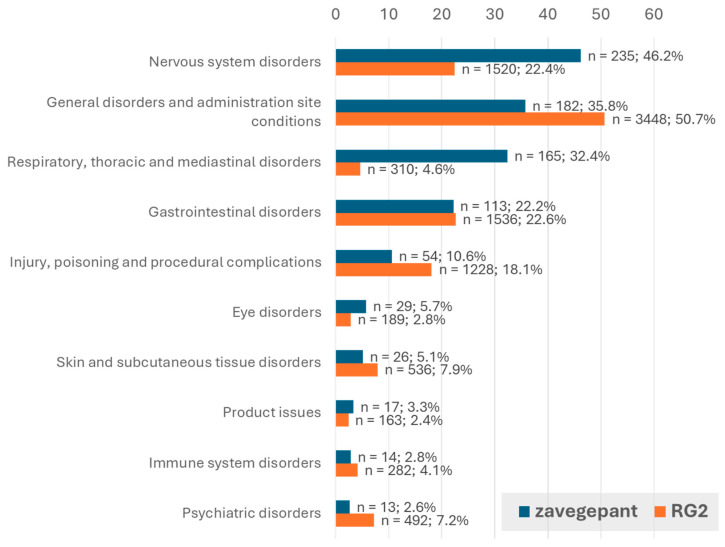
Adverse event distribution by the Medical Dictionary for Regulatory Activities (MedDRA^®^) System Organ Classes. RG: reference group. System Organ Classes presenting an associated number of ICSRs for zavegepant < to 10, were excluded from the figure.

Considering the types of observed AEs, after regrouping at the System Organ Class (SOC) level of the Medical Dictionary for Regulatory Activities (MedDRA^®^), the most frequently reported AEs for zavegepant were nervous system disorders (*n* = 235; 46.2%), general disorders and administration site conditions (*n* = 182; 35.8%), respiratory, thoracic and mediastinal disorders (*n* = 165; 32.4%), gastrointestinal disorders (*n* = 113; 22.2%), injury, poisoning and procedural complications (*n* = 54; 10.6%), and eye disorders (*n* = 29; 5.7%) (see Figure 2 for further details).

### 2.2. Disproportionality Analysis

For this analysis, reporting odds ratios (RORs) with 95% confidence intervals (CIs) were used as measures of disproportionality. SDRs were deemed significant if the associated ROR 95% CI lower bound was >1, in the presence of ≥3 ICSRs; more details are provided in Section 4. Zavegepant-related ICSRs were compared with the primary reference group (all other drugs present in the database, reference group 1; RG1) and RG2 at the MedDRA^®^ Preferred Term (PT) level. Within the MedDRA SOC nervous system disorders, dysgeusia represented the SDR with the highest ROR in both comparisons (*n* = 147; ROR = 94.72; 95% CI [78.19, 114.75] vs. RG1; ROR = 85.86; 95% CI [57.74, 127.66] vs. RG2), followed by taste disorder (29; 53.85; [37.01, 78.36] vs. RG1; 27.32; [14.55, 51.31] vs. RG2). Additional SDRs included brain fog (5; 13.82; [5.73, 33.35] vs. RG1; 3.20; [1.20, 8.52] vs. RG2), and burning sensation (20; 10.02; [6.41, 15.67] vs. RG1; 11.54; [6.33, 21.04] vs. RG2).

In the SOC general disorders and administration site conditions, the SDR with the highest ROR against RG1 was therapeutic product effect incomplete (33; 13.39; [9.41, 19.06]), followed by drug ineffective (89; 3.80; [3.02, 4.78]) and illness (6; 2.29; [1.02, 5.12]). No PT within this SOC showed a significant SDR in the comparison with RG2.

The SOC respiratory, thoracic, and mediastinal disorders were characterized by the highest number of observed SDRs. In detail, nasal discomfort showed the highest ROR in both comparisons (62; 298.85; [228.91, 390.17] vs. RG1; 942.76; [130.42, 6814.93] vs. RG2), followed by rhinalgia (10; 126.09; [67.34, 236.09] vs. RG1; 136.21; [17.40, 1066.22] vs. RG2). Additional SDRs observed versus RG1 included pharyngeal ulceration (3; 79.20; [25.42, 246.75]), upper-airway cough syndrome (16; 62.87; [38.19, 103.49]), sinus pain (4; 60.75; [22.69, 162.65]), throat irritation (49; 40.42; [30.10, 54.27]), oropharyngeal discomfort (7; 26.87; [12.74, 56.67]). Several of these SDRs were also confirmed versus RG2, including sinus pain (4; 17.94; [4.00, 80.38]), throat irritation (49; 40.12; [23.18, 69.43]), oropharyngeal discomfort (7; 13.53; [4.73, 38.73]), paranasal sinus discomfort (3; 20.15; [3.36, 120.87]), sinus congestion (3; 13.43; [2.70, 66.71]), oropharyngeal pain (18; 12.42; [6.53, 23.63]), sneezing (4; 10.76; [2.88, 40.20]), rhinorrhoea (10; 17.01; [6.68, 43.29]), epistaxis (11; 8.32; [3.91, 17.71]), and nasal congestion (6; 9.00; [3.19, 25.39]).

Among gastrointestinal disorders, the highest RORs were observed for retching (5; 7.82; [3.24, 18.87] vs. RG1; 4.81; [1.73, 13.41] vs. RG2), oral discomfort (3; 7.31; [2.35, 22.74] vs. RG1; 3.66; [1.02, 13.16] vs. RG2), and vomiting (48; 3.86; [2.87, 5.20] vs. RG1; 3.13; [2.26, 4.34] vs. RG2). Swollen tongue (3; 3.32; [1.07, 10.33]) and nausea (56; 2.66; [2.02, 3.51]) showed SDRs only in the comparison with RG1.

Within eye disorders, lacrimation increased was the SDR with the highest ROR value (*n* = 9; ROR = 10.54; 95% CI [5.45, 20.38] vs. RG1; ROR = 61.16; 95% CI [13.18, 283.83] vs. RG2), followed by photophobia (*n* = 3; ROR = 5.60; 95% CI [1.80, 17.42] vs. RG1), eye irritation (*n* = 7; ROR = 4.97; 95% CI [2.36, 10.48] vs. RG1; ROR = 13.53; 95% CI [4.73, 38.73] vs. RG2), and ocular hyperaemia (*n* = 4; ROR = 3.00; 95% CI [1.12, 8.02] vs. RG1; ROR = 7.68; 95% CI [2.24, 26.32] vs. RG2). Further details regarding the observed SDRs are available in Table 2.

## 3. Discussion

### 3.1. Descriptive Analysis

As for the descriptive analysis, a higher frequency of female than male patients was observed in ICSRs involving zavegepant as a suspected drug. This pattern aligns with what was observed in RG2 ICSRs. However, this difference is likely influenced by the well-established higher prevalence of migraine in women [27]. In terms of age distribution, the observed median age in zavegepant ICSRs was lower than that observed in RG2. However, the vast majority of zavegepant ICSRs did not report the patient’s age. Thus, this finding should be interpreted with caution. Indeed, the lower observed median age might reflect the status of zavegepant as a newly approved drug. This might be in line with common practice in clinical settings, in which it is frequent to reserve newly approved treatment options for patients who are typically more resilient or less complex to treat [28]. Nevertheless, considering the high percentage of missing data, the available age data may not be representative of the overall zavegepant-related ICSR population. This lack of data may be related to the drug’s early post-marketing phase, during which the timeliness of reporting is often prioritized over comprehensive completion of the ICSR [29]. This is also in line with the absolute predominance of ICSRs reporting only non-serious AEs. Indeed, in the early phases of commercialization, heightened attention often leads to reporting of any event potentially attributable to the drug, regardless of its seriousness [30]. However, the impact of the large proportion of ICSRs submitted through pharmaceutical companies, which have been shown to be less complete than those issued by regulatory structures, cannot be ruled out [31]. As far as ICSRs describing serious AEs were concerned, in eight cases, TTO data highlighted an onset in less than 24 h, and in six cases, a positive dechallenge was described. These data, although severely limited, might be viewed as consistent with the acute nature of zavegepant treatment. Furthermore, the positive dechallenge data seem to point towards at least an improvement in 20% of serious ICSRs.

### 3.2. Disproportionality Analysis

About the disproportionality analysis, within the MedDRA^®^ SOC nervous system disorders, the AE dysgeusia and taste disorder had the highest ROR values. Dysgeusia has already been described in association with CGRP-RAs, including zavegepant, in both pre- and post-marketing settings [32,33,34,35]. This AE may be linked to the role of CGRP in gustatory signal modulation. Animal studies have shown that CGRP receptors are expressed on presynaptic type III cells in taste buds. In this context, CGRP acts as a negative modulator of taste transmission [36]. Accordingly, inhibition of the CGRP receptor by CGRP-RAs could interfere with this pathway, leading to altered taste perception, often described as bitter or metallic [26]. In this context, the intranasal administration of zavegepant may represent an important factor in explaining observed disproportionality when comparing zavegepant ICSRs with the RG2. Indeed, although zavegepant reaches lower systemic concentrations than other CGRP-RAs [37], intranasal administration may increase direct exposure of taste buds to the drug [38]. Taste disorders such as dysgeusia, although generally non-serious, should not be underestimated. These AEs may negatively affect patients’ quality of life in several ways. For instance, dysgeusia can prompt individuals to increase consumption of sugary foods or beverages to mask the unpleasant taste [39]. This may complicate the management of diabetes and hypertension [40,41]. Moreover, given their unpleasant nature, these AEs may lead patients to delay drug administration, as observed for other drug classes [42]. This is particularly relevant because timely administration is crucial for acute migraine treatment, and postponement could reduce treatment effectiveness [43]. However, an important factor to consider regarding taste alterations is acute medication overuse among migraine-affected patients. Indeed, this condition is a well-characterized issue in acute migraine treatment, presenting in a non-negligible portion of patients [44]. Furthermore, taste disorders have been found to be associated with a large number of different drug classes, including triptans, NSAIDs, antiemetics, and other gepants within short time windows [45]. Therefore, in patients with frequent use of acute medications, taste alterations may not be attributable to a single agent alone but may reflect overall medication burden and repeated drug exposure. This further highlights the importance of longitudinal monitoring of prescriptions and concomitant treatments in this population.

Other AEs relative to the same SOC that were found to be disproportionate included alterations in consciousness, such as brain fog. It should be noted that these kinds of manifestations are among the most common symptoms of migraine attacks in their early phases [46]. However, the SDR for brain fog remained significant when zavegepant-related ICSRs were compared with RG2. This finding may be partly explained by differences in temporal attribution. As previously noted, zavegepant reaches peak plasma concentration more rapidly than the RG2 CGRP-RAs [32,37]. This difference may favor its use in closer temporal proximity to the onset of cognitive symptoms such as brain fog. This may reinforce the perceived temporal relationship between drug administration and symptoms occurring shortly thereafter. Indeed, brain fog can occur during the ictal and persist into the postdromal phases of migraine [47,48]. Therefore, it might be more readily attributed to zavegepant increasing its likelihood of reporting in related ICSRs. However, the small number of cases describing brain fog as an AE and the large number of reported lack-of-effectiveness cases limit our capacity to draw conclusions on the matter. Considering this, further focused investigations might be necessary.

As far as the SOC general disorders and administration site conditions were concerned, the SDRs observed for “therapeutic product effect incomplete” as well as “drug ineffective” when compared to RG1 require some consideration. In the context of migraine treatment, the disease itself could represent an important influencing factor. Indeed, the literature sources highlight how 30 to 40% patients treated with CGRP-RAs fail to get meaningful relief for each given attack [49]. However, these data are in line with other treatment options, such as triptans [50], hinting towards the presence of non-treatment-related variables [51]. Thus, in the absence of attack-level denominator data, no assumptions could be made in this sense.

Considering the SOC respiratory, thoracic, and mediastinal disorders, several PTs showed significant disproportionalities versus both RGs. Among the ones regarding the nasal mucosa, nasal discomfort was the only one reported in the full prescribing information for zavegepant [32]. Although conditions such as epistaxis and rhinorrhoea were not described in the full prescribing information, in the latter case, some evidence regarding its possible onset was reported in a phase 2/3 trial [26]. Such AEs are physiologically plausible, given potential local inhibition of CGRP-mediated neurotransmission. Indeed, the nasal and paranasal sinus mucosae are densely innervated by sensory fibers of the trigeminal nerve [52]. In these mucosae, CGRP plays a key homeostatic role by maintaining mucosal blood flow and protecting the epithelium. Thus, local inhibition of CGRP receptors may therefore interfere with physiological neurovascular regulation, leading to irritative or painful phenomena perceived at the level of the nasal mucosa [53]. Such local neurovascular interference may also indirectly promote the onset of AEs such as epistaxis. Indeed, sensations of dryness at the level of the nasal mucosa might, in turn, prompt patients to apply further mechanical stress to the tissue. However, to the best of our knowledge, no studies have specifically investigated these effects to date. Considering the growing relevance of the intranasal route for drug delivery in central nervous system disorders, further research in this area may therefore be warranted.

Nevertheless, these findings require careful interpretation. The disproportionalities observed for these AEs relative to RG2 may be influenced by differences in the route of administration, as second-generation CGRP-RAs are available only as oral formulations, whereas zavegepant is administered intranasally. Consequently, a higher reporting frequency of nasal-specific AEs would be anticipated and should be considered within the context of this intrinsic difference between the treatments.

Moreover, the remarkably elevated ROR estimates should be interpreted cautiously. Reporting imbalance may have substantially contributed to these findings, particularly for easily recognizable local events such as nasal discomfort. Given the relatively small number of reports, preferential reporting of these AEs may have amplified their relative contribution, leading to an overestimation of the observed disproportionality signals.

In addition to nasal AEs, several SDRs reported AEs involving the oropharyngeal region were also identified. Among those, some, such as oropharyngeal pain, upper airway syndrome with cough and throat irritation, were not reported in the FDA full prescribing information for zavegepant. However, as far as the latter two were concerned, evidence regarding the possibility of their onset was presented in the result of a previously cited phase 2/3 trial [26]. Considering the possible mechanisms at the basis of these AEs, it should be noted that CGRP plays a relevant modulatory role at the oropharyngeal level. Indeed, with its release by sensory afferent fibers of the trigeminal, facial, glossopharyngeal, and vagus nerves, CGRP contributes to both the regulation of swallowing and cough mechanisms [53,54]. Data from a relatively recent study showed that older patients with oropharyngeal dysphagia had markedly lower salivary CGRP concentrations than healthy controls, together with a significantly increased oropharyngeal sensory threshold and delayed vestibular-laryngeal closure [55]. It is therefore plausible that local inhibition of CGRP receptors may interfere with these regulatory circuits, leading to discomfort or pain in this region. Moreover, this inhibition may favor the development of other disproportionally reported AEs, namely the upper airway syndromes with cough-related manifestations. These manifestations may represent a non-negligible issue in patients predisposed to headache, such as those with migraine. Physical stimuli are known to exacerbate migraine attacks [56]. Although the available literature on this specific aspect is limited, one study of 38 patients found that 53% reported cough as a triggering or aggravating factor for migraine attacks [57]. Considering these findings, further studies on this topic may be of interest. Also, in this case, the influence of the route of administration on the RG2 disproportionalities and the possibility of confounding by route of administration should not be underestimated. Indeed, intranasal administration might increase exposure of the oropharyngeal mucosa compared with oral formulations [58], thus increasing the chance of onset of these AEs.

Considering gastrointestinal disorder-related AEs, vomiting and retching were disproportionately reported when zavegepant-related ICSRs were compared with both reference groups, whereas nausea showed an SDR only in comparison with RG1. These AEs were already partially associated with the drug in pre-marketing studies [32]. However, their interpretation should also consider the real-world context of acute migraine treatment. Retching, vomiting, and nausea are, in fact, closely related to the disease itself, representing common features of migraine. Furthermore, the onset of these AEs might also be influenced by the co-administration of drugs frequently used by patients affected by migraine (i.e., nonsteroidal anti-inflammatory drugs and triptans) [59,60]. These aspects cannot be adequately explored in an AEMS-based pharmacovigilance study. Information on timing, dose, treatment sequence, and actual co-administration is often lacking, especially for concomitant drugs. Therefore, focused studies addressing concomitant medication use, treatment timing, and overall acute medication burden in patients treated with zavegepant are warranted.

Finally, significant SDRs were observed for several AEs within the SOC eye disorders, such as increased lacrimation and eye irritation, compared to both RGs. These disproportionalities may again be related to both the role of CGRP at the ocular level and the peculiar route of administration of zavegepant. At the corneal level, CGRP-containing fibres with neurotrophic, vasomodulatory, and anti-inflammatory functions are distributed from the stroma to the epithelium [61]. Since the nasal and ocular mucosae share the same sensory innervation through the ophthalmic branch of the trigeminal nerve, which conveys nociceptive and autonomic signals from both the nasal cavities and periorbital structures, ocular AEs may result from reflex activation of shared trigeminal circuits. As previously noted, intranasal administration of zavegepant should be carefully considered when comparing zavegepant-related ICSRs with RG2. Indeed, theoretical nasolacrimal diffusion phenomena might increase drug exposure to the ocular mucosa, thereby increasing the likelihood of reporting these AEs [62]. AEs involving the ocular mucosa should be carefully monitored in patients with migraine. Indeed, ocular irritation may facilitate, in predisposed individuals, the onset of headache forms secondary to ocular strain [63]. Moreover, these conditions may directly impair patient functioning, particularly in occupational settings characterized by intense light exposure, which may further worsen these symptoms [64].

### 3.3. Strengths and Limitations

This study draws on real-world evidence from the AEMS database and offers additional information on the safety profile of zavegepant. Because zavegepant is the first CGRP-RA approved for intranasal use, its post-marketing safety characteristics are of particular interest. Although some previous real-world studies regarding CGRP-RAs are available [65,66], to the best of our knowledge, this is the first large-scale pharmacovigilance study to compare real-world safety data for zavegepant with those of other CGRP-RAs approved for the acute treatment of migraine. Although these results add a novel perspective on zavegepant-associated AEs, they should be interpreted with appropriate caution in view of several important limitations.

AEMS data are based on spontaneously reported ICSRs, and this type of reporting is inherently subject to duplicate submissions, inaccuracies, and considerable variability in the completeness and quality of the information provided. These issues were considered during the analysis, and a conservative approach was applied when information was limited. A relevant example is age data, which were not reported in the majority of zavegepant-related ICSRs. This substantially limits the interpretation of age-related patterns and comparisons with RG2, as the available age data may not be representative of the overall zavegepant-related ICSR population. Additional constraints specific to AEMS involved TTO, dechallenge, and rechallenge data, which were available for only a small proportion of serious ICSRs and were provided on a case-by-case basis, thereby limiting a robust assessment of these aspects. In addition to that, as explained in Section 3, the AEMS platform does not provide true outcome data, with “outcome” fields reporting the type of seriousness of the observed AE. Thus, the lack of data regarding AEs progression limits our considerations in this context. Furthermore, although literature-derived reports were excluded as a measure to limit potential duplicate reporting, as described in Section 4, this approach may have led to the omission of some clinically detailed ICSRs concerning rare or serious AEs.

Disproportionality analyses are also vulnerable to several well-known sources of bias, including notoriety bias and confounding by indication [67,68]. To reduce the potential effect of notoriety bias in our study, a caution-oriented approach was used when assessing AEs that are more likely to be affected by this phenomenon. Confounding by indication could also represent an important variable when interpreting ICSRs related to migraine treatments. To mitigate this issue, two main measures were taken. The first was to include two RGs, one of which consisted of drugs sharing the same mechanism of action as zavegepant and approved for the same therapeutic indication. The second was to exclude all ICSRs presenting as AEs, PTs directly referring to migraine. Even so, given the complex and variable clinical manifestations of migraine, the findings still require careful interpretation. Furthermore, the fact that second-generation CGRP-RAs are only administered orally might have introduced confounding by route of administration into the disproportionality results, especially for nasal and oropharyngeal AEs. For this reason, these findings were interpreted cautiously throughout the manuscript, detailing the influence of possible route-specific conditions on their onset.

Moreover, the choice to exclude ICSRs related to the use of CGRP-RAs in migraine prevention may have reduced the generalizability of our results and decreased the data-retrieval capacity of our study, as indication coding in spontaneous reporting systems can be incomplete or inconsistent. However, acute and preventive migraine treatment settings differ in several aspects (e.g., treatment goals, dosing patterns, duration of exposure, and patient characteristics). Therefore, including reports related to migraine prevention could have introduced additional heterogeneity and increased the risk of indication-related confounding. Accordingly, the findings of this study should be interpreted in the context of acute migraine treatment, while further studies are warranted to better characterize the safety of CGRP-RAS used for migraine prevention.

In addition, pharmacovigilance data should always be interpreted with caution, given additional methodological limitations, such as under-reporting relative to the overall treated population and the difficulty of fully accounting for confounding variables. As a result, the AEs captured in this database likely reflect only a fraction, and possibly an underestimate, of the AEs occurring in routine clinical practice. In addition, because the number of patients exposed to these drugs during the study period is unknown, it is not possible to derive incidence rates or calculate incidence fractions.

## 4. Materials and Methods

To achieve the objective of the study, a retrospective pharmacovigilance disproportionality analysis using spontaneous reporting data available from the FDA AEMS database was performed.

### 4.1. Data Source

AEMS is a widely used, publicly available repository that contains more than 30 million ICSRs submitted by consumers, healthcare professionals, and pharmaceutical companies from the United States as well as other regions, including Europe and Asia. In addition to unique case identifiers, these reports may include key details such as submission dates and AE onset dates, the reporting country, the qualification of the primary reporter, consumer characteristics (e.g., sex, age, and body weight), suspected and concomitant medicines with their indications, descriptions of the reported AEs, and information on AE seriousness [67,69]. AEMS was selected as the data source on the basis of its large-scale nature, combined with the possibility of accessing raw quarterly data files. Although requiring dedicated preprocessing steps, these characteristics allow for greater analytical flexibility than other platforms [70]. These features make AEMS a valuable tool for pharmacovigilance analyses, particularly for newly marketed drugs for which real-world safety evidence remains limited.

### 4.2. Database Pre-Processing

ICSR data were retrieved from the quarterly AEMS ASCII datasets publicly released by the FDA (accessed 9 April 2026). Files covering the period from Q1 2004 to Q4 2025 were downloaded and processed prior to analysis. When multiple versions of the same ICSR were available, only the most recent record was retained. Furthermore, reports originating from scientific literature were excluded. This latter exclusion was performed in order to focus on ICSRs deriving from spontaneous reporting only, and to further reduce the possibility of duplicate ICSRs, which are known to affect the AEMS platform [71,72]. Additionally, ICSRs lacking a meaningful AE description (e.g., containing only entries such as “unevaluable event” or “no adverse event”) were not considered. Potential duplicates were identified by checking key fields, including event dates, sex, age, reporting country, body weight, reported AE, and suspected active substances. Entries identified as probable duplicates were then removed. All reported AEs were coded using MedDRA^®^ version 28.1. MedDRA^®^ is developed under the supervision of the International Council for harmonization of Technical Requirements for Pharmaceuticals for Human Use. MedDRA^®^ is a multiaxial hierarchical terminology structured across five levels, spanning from highly specific, lowest-level terms to broader SOCs. In the present analysis, AEs were initially aggregated at the SOC level and subsequently evaluated at the PT level [73].

### 4.3. Extraction Criteria

ICSRs presenting zavegepant as a suspect drug (both primary and secondary) were extracted and analyzed. In addition to uniquely identifiable ICSRs presenting as suspect drugs, the other CGRP-RAs currently approved by the FDA for the acute treatment of migraine (i.e., ubrogepant and rimegepant, RG2) were also considered. ICSRs related to these latter drugs were extracted to compare their characteristics with those of zavegepant-related ICSRs. The decision to include both ICSRs were the CGRP-RAs were deemed as primary or secondary suspects was made in light of zavegepant’s newly marketed status. Indeed, limiting the analysis to primary suspects, given the overall contained number of ICSRs, could have reduced the impact of SDRs and led to the exclusion of potentially informative reports.

To maintain uniformity across both ICSR groups with respect to the use indication, ICSRs reporting migraine prevention as the indication for use for the CGRP-RAs in RG2 were also removed. Indeed, atogepant is approved only for prophylactic treatment, while rimegepant is approved for both acute and prophylactic migraine treatment. Furthermore, to reduce confounding by indication, all ICSRs reporting AEs with PTs explicitly referring to migraine (e.g., migraine, migraine with aura, migraine without aura) were also removed from both groups (full details are available in Supplementary Table S4). No restrictions based on gender or age group were reported in the ICSRs, nor were any restrictions applied regarding the geographical origin of the ICSRs themselves.

### 4.4. Data Analyses

A descriptive statistical analysis was conducted to examine the demographic and clinical characteristics of AEMS ICSRs for zavegepant compared with those for other CGRP-RAs. This analysis encompassed patient characteristics (such as gender and age), primary sources of information, year of reporting, reporting countries, and the AE seriousness. Continuous variables were presented as medians with associated quartile 1–quartile 3 (Q1–Q3), while categorical variables were expressed as absolute values with relative percentages. Serious ICSRs were considered as such if presenting at least a serious AE, which, in accordance with guidelines from regulatory agencies, was defined as an AE that “results in death, is life-threatening, requires hospitalization or prolongation of existing hospitalisation, results in persistent or significant disability or incapacity, or is a birth defect” [74,75]. In addition to that, an AE was classified as serious if reported as an “other serious important medical event” in the examined ICSR [75].

Considering case-by-case assessment, TTO, dechallenge, and rechallenge data were evaluated when available. On the basis of previously published articles [76], the TTTO was evaluated as the difference in days between the therapy initiation and the first AE onset.

The disproportionality measure used to evaluate the presence of eventual SDRs was the ROR with the corresponding 95%CI. The statistical significance threshold was set at values >1 of the lower ROR 95% CI limit, with a minimum of three ICSRs for each drug–event pair. To evaluate significant disproportionalities in the odds of AE, a two-step process was implemented: first, a disproportionality analysis was conducted comparing zavegepant-related ICSRs with those relative to all other drugs in the database (RG1). Then the comparison was restricted only to ICSRs presenting other CGRP-RAs approved for the acute treatment of migraine as a suspected drug (reference group 2, RG2). The expectedness of the reported AEs for zavegepant was evaluated using data from the US FDA Full Prescribing Information. A careful clinical-based approach was adopted when interpreting the Full Prescribing Information. Broadly encompassing terms, such as “taste disorder,” were considered as umbrella terms for more specific PTs, such as “dysgeusia”. Furthermore, conditions that share a close relationship with core migraine symptoms, such as brain fog, were considered as possibly related to the disease and thus known. Full details available in Supplementary Table S3.

All statistical analyses were performed using the R software (version 4.5.3, R Core Team (2023), R Foundation for Statistical Computing, Vienna, Austria) and the RStudio working environment (version 2026.01; Posit team (2025), Posit Software, PBC, Boston, MA, USA). The present study is reported in accordance with the “The REporting of A Disproportionality Analysis for DrUg Safety Signal Detection Using Individual Case Safety Reports in PharmacoVigilance” (READUS-PV) statement [77]. The READUS-PV checklist is available in the Supplementary Table S2.

## 5. Conclusions

The findings of this study depict an AE profile for zavegepant consistent with what is already known for other CGRP-RAs and with the drug’s mechanism of action. Although most of the observed AEs appear to be non-serious, their occurrence may still have a relevant impact on patients’ quality of life. In addition, the onset of some of these AEs may induce a delay or discourage timely drug administration, thereby reducing the potential benefit of these treatments. Considering this, further studies using appropriate confirmatory methods are needed to better define the incidence and clinical relevance of these AEs, their potential long-term impact, and their influence on treatment adherence and effectiveness.

## Figures and Tables

**Table 1:** Demographic and adverse event characteristics of zavegepant-related ICSRs.

Characteristic	Zavegepant-Related ICSRs *n* = 509 (%)	Other CGRP-RA-Related ICSRs *n* = 6798 (%)
**Sex** **, *n* (%)**		
Women	353 (69.4)	4574 (67.3)
Men	43 (8.4)	867 (12.8)
Not Available	113 (22.2)	1357 (20.0)
**Age, median (Q1–Q3)**	45 (34–56)	50 (37–61)
**Age group, *n* (%)**		
0–17 years	2 (0.4)	141 (2.1)
18–34 years	46 (9.0)	622 (9.1)
35–64 years	117 (23.0)	2312 (34.0)
65–80 years	16 (3.1)	648 (9.5)
>80 years	0 (0.0)	62 (0.9)
Not available	328 (64.4)	3013 (44.3)
**Reporter type, *n* (%)**		
Patient/consumer	208 (40.9)	5254 (77.3)
Other healthcare professionals	147 (28.9)	764 (11.2)
Physician	148 (29.1)	621 (9.1)
Pharmacist	6 (1.2)	127 (1.9)
Not specified	0 (0.0)	32 (0.5)
**Seriousness, *n* (%)**		
Serious	30 (5.9)	1298 (19.1)
Not serious	479 (94.1)	5500 (80.9)
**Outcome, *n* (%)**		
Death	0 (0.0)	84 (1.2)
Disability	0 (0.0)	60 (0.9)
Hospitalization	5 (1.0)	232 (3.4)
Congenital anomaly	0 (0.0)	11 (0.2)
Life-threatening	1 (0.2)	45 (0.7)
Other serious medical event	24 (4.7)	853 (12.5)
Required intervention	0 (0.0)	13 (0.2)
Not serious	479 (94.1)	5500 (80.9)

Q1: first quartile; Q3: third quartile; ICSR: individual case safety report; CGRP-RA: calcitonin gene-related peptide receptor antagonist.

**Table 2:** Disproportionality analysis and expectedness evaluations for zavegepant ICSRs.

MedDRA PT by SOC	Zavegepant ICSRs *	ROR [95% CI] vs. RG1	ROR [95% CI] vs. RG2	Expected #
**Nervous system disorders**				
dysgeusia	147	94.72 [78.19, 114.75]	85.86 [57.74, 127.66]	YES (taste disorders)
taste disorder	29	53.85 [37.01, 78.36]	27.32 [14.55, 51.31]	YES (taste disorders)
brain fog	5	13.82 [5.73, 33.35]	3.20 [1.20, 8.52]	YES (migraine-related)
burning sensation	20	10.02 [6.41, 15.67]	11.54 [6.33, 21.04]	NO
head discomfort	5	9.64 [3.99, 23.26]	1.81 [0.71, 4.63]	YES (migraine-related)
hypoaesthesia	7	1.56 [0.74, 3.29]	1.38 [0.63, 3.02]	NO
headache	23	1.28 [0.84, 1.94]	0.67 [0.44, 1.03]	YES (migraine-related)
dizziness	14	0.95 [0.56, 1.62]	0.61 [0.35, 1.05]	NO
paraesthesia	4	0.86 [0.32, 2.30]	0.59 [0.22, 1.61]	NO
somnolence	3	0.54 [0.17, 1.68]	0.21 [0.07, 0.66]	NO
**General disorders and administration site conditions**				
therapeutic product effect incomplete	33	13.39 [9.41, 19.06]	1.26 [0.87, 1.82]	YES
drug ineffective	89	3.80 [3.02, 4.78]	0.68 [0.54, 0.86]	YES
illness	6	2.29 [1.02, 5.12]	1.06 [0.46, 2.45]	NO
swelling face	4	2.08 [0.78, 5.56]	1.67 [0.59, 4.74]	YES
malaise	10	0.80 [0.43, 1.50]	1.20 [0.62, 2.31]	NO
feeling abnormal	4	0.55 [0.21, 1.47]	0.25 [0.09, 0.68]	NO
pain	8	0.48 [0.24, 0.97]	0.89 [0.43, 1.83]	NO
fatigue	7	0.30 [0.14, 0.63]	0.44 [0.21, 0.94]	NO
**Respiratory, thoracic, and mediastinal disorders**				
nasal discomfort	62	298.85 [228.91, 390.17]	942.76 [130.42, 6814.93]	YES
rhinalgia	10	126.09 [67.34, 236.09]	136.21 [17.40, 1066.22]	NO
pharyngeal ulceration	3	79.20 [25.42, 246.75]	-	NO
upper-airway cough syndrome	16	62.87 [38.19, 103.49]	-	NO
sinus pain	4	60.75 [22.69, 162.65]	17.94 [4.00, 80.38]	NO
throat irritation	49	40.42 [30.10, 54.27]	40.12 [23.18, 69.43]	NO
oropharyngeal discomfort	7	26.87 [12.74, 56.67]	13.53 [4.73, 38.73]	NO
paranasal sinus discomfort	3	26.56 [8.53, 82.67]	20.15 [3.36, 120.87]	NO
pharyngeal swelling	3	11.14 [3.58, 34.66]	1.83 [0.55, 6.13]	NO
sinus congestion	3	7.20 [2.31, 22.40]	13.43 [2.70, 66.71]	NO
oropharyngeal pain	18	6.64 [4.15, 10.63]	12.42 [6.53, 23.63]	NO
sneezing	4	6.02 [2.25, 16.10]	10.76 [2.88, 40.20]	YES
rhinorrhoea	10	5.27 [2.82, 9.86]	17.01 [6.68, 43.29]	NO
epistaxis	11	5.24 [2.88, 9.52]	8.32 [3.91, 17.71]	NO
nasal congestion	6	3.43 [1.53, 7.67]	9.00 [3.19, 25.39]	NO
dyspnoea	7	0.42 [0.20, 0.89]	0.75 [0.35, 1.61]	NO
**Gastrointestinal disorders**				
retching	5	7.82 [3.24, 18.87]	4.81 [1.73, 13.41]	NO
oral discomfort	3	7.31 [2.35, 22.74]	3.66 [1.02, 13.16]	NO
vomiting	48	3.86 [2.87, 5.20]	3.13 [2.26, 4.34]	YES
swollen tongue	3	3.32 [1.07, 10.33]	1.29 [0.39, 4.23]	NO
nausea	56	2.66 [2.02, 3.51]	1.06 [0.79, 1.41]	YES
abdominal discomfort	7	1.43 [0.68, 3.02]	0.74 [0.34, 1.59]	NO
dysphagia	3	1.07 [0.34, 3.33]	1.29 [0.39, 4.23]	NO
abdominal pain upper	5	0.83 [0.34, 2.00]	0.37 [0.15, 0.90]	NO
**Injury, poisoning, and procedural complications**				
wrong technique in device usage process	4	3.81 [1.42, 10.19]	-	NO
wrong technique in product usage process	4	0.61 [0.23, 1.63]	0.90 [0.33, 2.49]	NO
**Eye disorders**				
lacrimation increased	9	10.54 [5.45, 20.38]	61.16 [13.18, 283.83]	NO
photophobia	3	5.60 [1.80, 17.42]	2.68 [0.77, 9.29]	YES (migraine-related)
eye irritation	7	4.97 [2.36, 10.48]	13.53 [4.73, 38.73]	NO
ocular hyperaemia	4	3.00 [1.12, 8.02]	7.68 [2.24, 26.32]	NO
eye swelling	3	2.70 [0.87, 8.40]	3.09 [0.88, 10.88]	NO
eye pain	3	1.91 [0.61, 5.94]	1.61 [0.48, 5.35]	NO
vision blurred	3	0.75 [0.24, 2.33]	0.98 [0.30, 3.18]	NO
**Skin and subcutaneous tissue disorders**				
skin burning sensation	3	1.60 [0.51, 4.98]	3.09 [0.88, 10.88]	YES
erythema	6	0.99 [0.44, 2.21]	2.99 [1.23, 7.28]	YES
pruritus	7	0.62 [0.29, 1.31]	0.66 [0.31, 1.42]	YES
rash	7	0.56 [0.27, 1.18]	0.55 [0.26, 1.18]	YES
**Immune system disorders**				
anaphylactic reaction	3	2.18 [0.70, 6.78]	1.29 [0.39, 4.23]	YES (hypersensitivity)
drug hypersensitivity	9	1.93 [1.00, 3.73]	1.66 [0.83, 3.34]	YES (hypersensitivity)
**Product issues**				
product tastes abnormal	12	31.12 [17.55, 55.19]	4.80 [2.47, 9.33]	NO
**Musculoskeletal and connective tissue disorders**				
neck pain	7	4.18 [1.98, 8.81]	3.04 [1.33, 6.94]	NO
**Ear and labyrinth disorders**				
ear discomfort	3	9.57 [3.08, 29.78]	2.87 [0.82, 10.02]	NO
**Vascular disorders**				
hypertension	3	0.50 [0.16, 1.56]	0.70 [0.22, 2.24]	YES
**Infections and infestations**				
nasopharyngitis	3	0.56 [0.18, 1.74]	2.87 [0.82, 10.02]	NO

Abbreviations: MedDRA: Medical dictionary for regulatory activities; PT: Preferred Term; RG: reference group; RG1: reference group 1 (all other drugs present in the AEMS database); RG2: reference group 2 (ubrogepant and rimegepant for acute migraine treatment); ROR: reporting odds ratio; SOC: system organ class. * Values in descending order based on SOC frequency of observation and ROR results when comparing zavegepant ICSRs to RG1. Preferred Terms with fewer than 3 associated ICSRs were excluded from the table. # Based on the FDA Full Prescribing Information for zavegepant. A conservative interpretation of the Full Prescribing Information was used for notoriety: broader terms (e.g., taste disorder) were considered as umbrella terms for more specific Preferred Terms (e.g., dysgeusia) and reported in round brackets, while symptoms closely linked to migraine itself (e.g., brain fog) were considered as possibly disease-related and therefore known. Full details available in Table S3.

## Data Availability

This study was entirely based on publicly anonymized data made available by the Food and Drug Administration. The raw data can be downloaded at the following link: https://fis.fda.gov/extensions/FPD-QDE-FAERS/FPD-QDE-FAERS.html (accessed on 8 April 2026).

## Supplementary Materials

The following supporting information can be downloaded at https://www.mdpi.com/article/10.3390/ph19060943/s1: Table S1: Geographical distribution of analyzed individual Case Safety Reports; Table S2: READUS-PV checklist; Table S3: Grouping categories used for adverse event expectedness evaluation; Table S4: Migraine-related Preferred Terms leading to zavegepant and RG2 related ICSR exclusion.

## Abbreviations

The following abbreviations are used in this manuscript:

AE/AEs	Adverse event(s)
AEMS	Adverse Event Monitoring System
CGRP	Calcitonin gene-related peptide
CGRP-RA/CGRP-RAs	Calcitonin gene-related peptide receptor antagonist(s)
CI/CIs	Confidence interval(s)
FDA	Food and Drug Administration
ICSR/ICSRs	Individual Case Safety Report(s)
MedDRA	Medical Dictionary for Regulatory Activities
PT/PTs	Preferred Term(s)
Q1–Q3	Quartile 1–quartile 3
READUS-PV	The REporting of A Disproportionality Analysis for DrUg Safety Signal Detection Using Individual Case Safety Reports in PharmacoVigilance
RG/RGs	Reference group(s)
ROR/RORs	Reporting odds ratio(s)
SDR/SDRs	Signal(s) of disproportionate reporting
SOC/SOCs	System Organ Class(es)
TTO	Time to onset
US/USA	United States/United States of America
