# Supplementary material for: Safety Profile of Zavegepant in the Treatment of Acute Migraine: Insights from the FDA Adverse Event Monitoring System Database
Source: Pharmaceuticals (Basel). 2026 Jun 15;19(6):943. doi: 10.3390/ph19060943 (PMC13304639; doi:10.3390/ph19060943)
Supplement: Supplementary file 1 [file pharmaceuticals-19-00943-s001.zip › pharmaceuticals-4343466-supplementary.pdf]

Table S1. Geographical distribution of analyzed individual case safety reports

Reporter country n (%)	Zavegepant-related ICSRs n = 509 (%)	Other CGRP-RA-related ICSRs n = 6798 (%)
Argentine Republic	0 (0.0)	2 (0.0)
Commonwealth of Australia	0 (0.0)	1 (0.0)
Republic of Austria	0 (0.0)	1 (0.0)
Kingdom of Belgium	0 (0.0)	3 (0.0)
Canada	0 (0.0)	21 (0.3)
Republic of Chile	0 (0.0)	11 (0.2)
People's Republic of China	0 (0.0)	7 (0.1)
Republic of Croatia	0 (0.0)	1 (0.0)
Czech Republic	0 (0.0)	1 (0.0)
Kingdom of Denmark	0 (0.0)	2 (0.0)
Republic of Finland	0 (0.0)	5 (0.1)
French Republic	0 (0.0)	8 (0.1)
Federal Republic of Germany	0 (0.0)	2 (0.0)
Hellenic Republic	0 (0.0)	2 (0.0)
Republic of Hungary	0 (0.0)	3 (0.0)
Ireland	0 (0.0)	1 (0.0)
State of Israel	0 (0.0)	8 (0.1)
Japan	0 (0.0)	1 (0.0)
Republic of Lithuania	0 (0.0)	1 (0.0)
Malaysia	0 (0.0)	2 (0.0)
United Mexican States	0 (0.0)	2 (0.0)
Kingdom of Norway	0 (0.0)	1 (0.0)
Republic of Peru	0 (0.0)	4 (0.1)
Republic of the Philippines	0 (0.0)	1 (0.0)
Republic of Poland	0 (0.0)	2 (0.0)
Portuguese Republic	0 (0.0)	5 (0.1)
Commonwealth of Puerto Rico	2 (0.4)	11 (0.2)
Romania	0 (0.0)	2 (0.0)
Kingdom of Saudi Arabia	0 (0.0)	2 (0.0)
Kingdom of Spain	0 (0.0)	7 (0.1)
Kingdom of Sweden	0 (0.0)	3 (0.0)
Republic of Turkey	0 (0.0)	1 (0.0)
United Arab Emirates	0 (0.0)	2 (0.0)
United Kingdom of Great Britain and Northern Ireland	0 (0.0)	45 (0.7)
United States of America	507 (99.6)	6609 (97.2)
Not available	0 (0.0)	18 (0.3)

ICSR: individual case safety report; CGRP-RA: calcitonin gene related peptide receptor antagonist

Table S2. READUS-PV checklist

Section and topic	Item #	Checklist item	Location where item is reported
**Title**			
	1a	*If disproportionality analyses are a prominent component of the published study, the study should be identified as a "disproportionality analysis". The type of data and name of the database(s) should be specified.*	Lines 1 - 3
	1b	*Report the name of adverse event(s) and/or drug(s) under study, when applicable.*	Lines 1 - 3
**Introduction**			
Background	*2a*	*Describe the drug(s) and its utilization, the nature of the adverse event(s) under study and its frequency, and the existing knowledge on the drug-event combination.*	Lines 75 -78
	2b	*Specify the rationale for performing the analysis, e.g., as part of routine pharmacovigilance, to investigate an overall safety profile, or to assess a pre-specified hypothesis.*	Lines 79 - 84
	2c	*Explain why ICSR databases and disproportionality analysis are suitable to fill the knowledge gap.*	Lines 84 - 87
Objectives	*3*	*State specific objectives, identifying the adverse event(s), the drug(s), and the reference group, including any pre-specified hypothesis, if applicable.*	Lines 88 - 90
**Methods**			
Study design	*4a*	*Identify the study (i.e., "disproportionality analysis") and the type of data used (e.g., "individual case safety reports").*	Lines 427 – 429<br>445 – 447
	4b	*Provide an outline of the entire study design, including primary and sensitivity analyses performed, and other designs such as case-by-case analysis or literature review.*	Lines 483 - 517
Data description, access, and pre-processing	*5a*	*Specify the name of the database(s), the database(s) custodian, and the coverage. Specify the type/number of drugs included within the database and the thesaurus, taxonomies, or ontologies used for coding drugs and events.*	Lines 431 - 438
	5b	*Specify the extraction dates and describe and justify all choices used for data pre-processing, including any data transformation or exclusion, if appropriate.*	Lines 445 - 461
Variables definition	*6a*	*Describe the study population, including any restriction.*	Lines 463 - 481
	6b	*Describe the nature and the meaning of key variables assessed in the work.*	Lines 482 - 518
	6c	*Specify and justify any grouping of drugs or events. For drugs, specify and justify whether active ingredients/trade names/salts were considered and/or the selected role.*	Lines 468 - 472
	6d	*Describe any additional data source used, the type of data, and how they interact with ICSRs.*	NA
Statistical methods	*7a*	*Present any descriptive analysis performed, specifying variables investigated, statistical tests, and significance thresholds.*	Lines 483 - 494
	7b	*Describe the measure(s) selected for the disproportionality analysis including any threshold used to identify signals of disproportionate reporting. Explain the reason for this choice if applicable.*	Lines 499 - 504
	7c	*Clearly describe any sensitivity analysis and any tool to control confounding, including any restriction, subgroup, stratification, adjustment, or interaction.*	Lines 474 – 479<br>505 – 506

	7d	*Specify the variables and methods used for the case-by-case analysis, including any algorithm or criteria used to assess causality, if performed.*	
	7e	*Specify any statistical methods used for other data sources.*	NA
**Results**			
Participants	*8a*	*Specify the number of individual case safety reports included at each stage, including reasons for exclusion.*	Lines 93 -101
	8b	*Provide key demographic and clinical characteristics of cases, if possible comparing cases with any appropriate reference group.*	Lines 106 - 117
Disproportionality analysis	*9*	*Present all results including confidence intervals. Present also results of sensitivity analyses, if performed.*	Lines 150 - 190
Case-by-case analysis	*10*	*Present the case-by-case analysis of key variables. Present the causality assessment, if applicable.*	Lines 130 -137
**Discussion**			
Key results	*11*	*Discuss key results with reference to study objectives and contextualize them within the current literature and other consulted sources. Clearly discriminate between expected reactions and emerging safety signals.*	Lines 201 - 366
External validity	*12a*	*Discuss the external validity of the results to the general population.*	Lines 239 -255<br>288 – 297<br>359 - 366
	12b	*Discuss the potential relevance of results in clinical practice*	Lines 253 -255<br>266 – 270<br>274 – 279<br>285 – 297<br>325 – 330<br>361 - 366
	12c	*Propose further study designs if applicable*	Lines 266 – 270<br>343 -348
Limitations	*13*	*Present general limitations, making clear that disproportionality analysis alone cannot prove causation or measure incidence, and specific limitations, including confounding and reporting bias and efforts to mitigate them.*	Lines 377 - 425
**Declarations**			
	14a	*Provide the source of funding/sponsorship and the role of the funders/sponsors for the present study and for any original study on which the present article is based.*	NA
	14b	*Clearly identify potential commercial and intellectual conflicts of interest (e.g., link to any drug/event investigated, whether financial, legal action, or software used).*	NA
	14c	*Declare any institutional approval needed or granted in the investigation.*	NA
	14d	*Include a statement on data availability, code availability (including the version of the statistical software used), and protocol registration.*	Lines 545 - 548

Table S3. Grouping categories used for adverse event expectedness evaluation

Grouping category	Regrouped preferred terms
**Taste disorders**	dysgeusia, taste disorder
**Migraine-related terms**	brain fog, head discomfort, hypoaesthesia, headache, photophobia
**Hypersensitivity**	drug hypersensitivity, anaphylactic reaction

Table S4. Migraine-related Preferred Terms leading to zavegepant and RG2 related ICSR exclusion

MedDRA® SOC	MedDRA® PT
Nervous system disorders	atypical migraine
	hemiplegic migraine
	migraine
	migraine postdrome
	migraine with aura
	migraine without aura
	ophthalmic migraine
	vestibular migraine

ICSR: individual case safety report; MedDRA®: Medical dictionary for regulatory activities; PT: preferred term; SOC: system organ class
